# Age-Related Decrease in Stress Responsiveness and Proactive Coping in Male Mice

**DOI:** 10.3389/fnagi.2018.00128

**Published:** 2018-05-07

**Authors:** Hee-Jin Oh, Minah Song, Young Ki Kim, Jae Ryong Bae, Seung-Yun Cha, Ji Young Bae, Yeongmin Kim, Minsu You, Younpyo Lee, Jieun Shim, Sungho Maeng

**Affiliations:** Graduate School of East-West Medical Science, Kyung Hee University, Yongin, South Korea

**Keywords:** age, corticosterone, stress responsiveness, proactive coping, active avoidance, glucocorticoid receptor

## Abstract

Coping is a strategic approach to dealing with stressful situations. Those who use proactive coping strategies tend to accept changes and act before changes are expected. In contrast, those who use reactive coping are less flexible and more likely to act in response to changes. However, little research has assessed how coping style changes with age. This study investigated age-related changes in coping strategies and stress responsiveness and the influence of age on the processing of conditioned fear memory in 2-, 12- and 23-month-old male mice. Coping strategy was measured by comparing the escape latency in an active avoidance test and by comparing responses to a shock prod. The results showed that proactivity in coping response gradually decreased with age. Stress responsiveness, measured by stress-induced concentration of corticosterone, was also highest in 2-month-old mice and decreased with age. Consolidation of fear memory was highest in 12-month-old mice and was negatively correlated with the degree of stress responsiveness and proactivity in coping. Fear extinction did not differ among age groups and was not correlated with stress responsiveness or the proactivity of coping. However, the maintenance of extinct fear memory, which was best in 2-month-old mice and worst in 12-month-old mice, was negatively correlated with stress responsiveness but not with coping style. Age-dependent changes in the expression of glucocorticoid receptor (GR) and its regulatory co-chaperones, which are accepted mechanisms for stress hormone stimulation, were measured in the hippocampus. The expression of GR was increased at 12 months compared to other age groups. There were no differences in Hsp70 and BAG1 expression by age. These results can be summarized as follows: (1) stress responsiveness and proactivity in coping decreased with age class; (2) consolidation of fear memory was negatively correlated with both stress responsiveness and proactivity; however, maintenance of extinct fear memory was negatively correlated with stress responsiveness only; and (3) consolidation and maintenance of extinct fear memory appeared to be more influenced by factors other than stress reactivity and proactivity in coping, such as the amount of hippocampal glucocorticoid expression.

## Introduction

Coping is a conscious effort to solve problems by minimizing stressful conflicts (Folkman, 1997). Coping strategies can be classified as reactive or proactive. A reactive coping response occurs after the stressor, while a proactive coping response aims to head off a future stressor (Koolhaas et al., 1999). It has been suggested that coping style explains vulnerability to stress-mediated diseases in animals (Koolhaas et al., 1999). Previous research has also identified age-related changes in personality traits, such as self-confidence, warmth, self-control and emotional stability (Roberts and Mroczek, 2008). Problem solving skills improve with age due to improved cognitive ability and social experiences (Leahy et al., 2017). In addition, older people are better at regulating their emotions, emphasizing positive emotions and curtailing negative ones, and negotiating effectively with others using relational management skills (Blanchard-Fields et al., 2004). These changes may be associated with improved coping skills. Although of great interest, age-related changes in coping style, impact on stress-related diseases and biological basis remain unknown.

This study investigated age-related changes in coping strategies and stress responsiveness and the influence of age on the processing of conditioned fear memory in mice. Stress responsiveness, coping style, conditioned fear memory regulation and the expression of glucocorticoid stress hormone-responding proteins in the hippocampus were investigated in three different age groups of mice. For the measurement of coping style, latency to avoid a noxious stimulus in an active avoidance test and the response to a shock prod were observed. For stress responsiveness, circulating corticosterone was measured at the circadian nadir, circadian peak and 2 h after immobilization stress. For fear regulation, the consolidation, extinction, and extinction retention of conditioned fear memory were measured. Then the expression levels of glucocorticoid receptor (GR), which have important regulatory roles on the hypothalamus-pituitary-adrenal (HPA) axis responsiveness and Hsp70 and BAG1, which are intracellular co-chaperones that regulate the activity of GR, have been determined in the hippocampus.

## Materials and Methods

### Animals

Male C57Bl/6 mice (Central Laboratory Animals Inc., South Korea; *n* = 104) were housed in a temperature- and humidity-controlled environment under light (7:00–19:00) and dark (19:00–7:00) cycle alternating every 12-h with free access to food and water.

All protocols were approved by the Institutional Animal Care and Use Community of Kyung Hee University (KHUAGC-16-19) and were conducted in accordance with the NIH Guide for the Care and Use of Laboratory Animals. Behavioral tests were conducted between the hours of 9:00 and 12:00 except fear extinction training which were conducted between 9:00 and 18:00.

### Active Avoidance

Escape latency in an active avoidance test was used to measure coping style, using the method of a previous report with slight modifications (González-Salinas et al., 2015). The test was performed in a two-compartment shuttle box with steel grids on the floor and a guillotine door separating the two compartments. Initially, with the house light and tone off and the guillotine door closed, an animal was placed into one of the compartments and left for 30 s. Then the house light and 2000 Hz 80 dB tone were turned on (conditioned stimulus; CS) and the guillotine door was opened. After 10 s, 0.45 mA electric foot shock (unconditioned stimulus; US) was applied for an additional 10 s. Immediately after the mouse entered the opposite compartment, the connecting door was closed, and both CS and US were discontinued. An inter-trial interval of variable duration (30–90 s) occurred between each session. During 20 repetitive sessions, the step-through latency to enter the opposite compartment was measured. If the mouse crossed within 10 s (before the presentation of US), its behavior was considered as “avoidance.” If it crossed within 10–20 s, its behavior was considered “escape.” Otherwise its behavior was classified as “fail” to escape, and latency was considered 20 s.

Seventy-two hours later, retention of avoidance memory was measured. As in the avoidance condition, mice were initially placed in one of the compartments for 30 s, and during the 10 s of CS and 10 s of CS+US presentation, step-through latency was measured.

### Hot Plate

Pain sensitivity can affect step-through latency in active avoidance. Therefore, a hot plate test was performed to compare pain thresholds by age. A mouse was placed on the surface of a hot plate apparatus set to 52°C. During the 60-s cut-off time, the latency to lick the hind paw or jump was recorded.

### Shock Prod Test

The test was performed in a plexiglas cage identical to the home cage of the mice. The floor was covered with sawdust, and a shock probe was inserted 1 cm above the sawdust through a small hole on the wall of the plexiglas cage. When the mouse touched the probe with its body, an electric current of 1.5 mA was delivered to the mouse. The behavioral response was recorded for 10 min and was classified into six elements: immobility, burying, rearing, exploring, grooming and rattling.

### Measurement of Serum Corticosterone

Blood was collected from the retro-orbital vein at 7:00 (nadir) and 19:00 (peak) to measure circadian changes in corticosterone concentration (Kakihana and Moore, 1976). To measure the change in cortisol concentration due to stress, restraint stress was applied for 2 h (9:00 AM–11:00 AM), and blood was collected from the retro-orbital vein. Serum was separated from the collected blood and stored frozen in a deep freezer until measurement. Serum corticosterone concentration was measured by a corticosterone ELISA kit (ADI-900-097, Enzo Life Sciences, Inc., Farmingdale, NY, USA), according to the manufacturer’s recommended methods. Restraint stress was applied by wrapping the body of mice with tape.

### Conditional Fear Response

Consolidation, extinction and extinction retention of conditioned fear were measured with the fear conditioning paradigm. For acquisition of fear memory, mice were placed in a plexiglass chamber (JD-SI-11, Jeungdo B&P, South Korea) for 3 min, and CS composed of 30 s of 80 dB tone and chamber illumination (CS) was delivered. An electrified foot shock (US) of 0.45 mA was delivered during the last 2 s of CS. Then, after an intertrial interval (ITI) of 30 s, the delivery of CS-US plus ITI was given four more times.

The next day, the freezing response of mice was measured in the chamber over 3 min to assess fear memory consolidation. For extinction training, CS without the accompanying US was delivered 30 times in the same chamber. For the measurement of fear memory extinction, the freezing response was measured for 3 min. On the following day, extinction retention was measured in the same chamber for 3 min. All activities were videotaped and analyzed by an unbiased reader. All of the behavioral measurements were conducted between 9:00–12:00 except the fear extinction training which were conducted from 9:00 to 18:00.

### Western Blot

For tissue collection, mice were anesthetized by an overdose of ether. From the brain, each hippocampus was rapidly dissected and stored at −80° until use. Immunoblotting was performed as described previously (Shin et al., 2014). In brief, the hippocampus was denatured in lysis buffer with a tissue grinder. After determination of the protein concentration of the homogenate, it was resolved by SDS-PAGE and transferred to the PVDF membrane. Primary antibodies (anti-GR; ab9568, Abcam, London, UK, anti-HSP70; #4872, Cell Signaling Tech, Danvers, MA, USA, anti-BAG1; sc-377454, Santa Cruz Biotech, Dallas, TX, USA) were diluted to a 1:1000 concentration ratio and applied overnight at 4°C. After an hour of incubation in 1:1000 diluted secondary antibody, bands were visualized by ECL solution (Thermo Fisher Scientific, Waltham, MA, USA) and quantified with a chemiluminiscence detector (Davinch Chemi Imaging System, CellTagen, South Korea) and the ImageJ image analysis software (NIH, Bethesda, MD, USA). Internal standard (a mixture of all samples in small portions) was used for comparison by loading onto every membrane.

### Statistical Analysis

SPSS ver. 20 (IBM, New York, NY, USA) was used for the statistical analysis. Between-group comparisons were made with one- and two-way ANOVAs. Then, Tukey’s HSD test was performed for significant results. Statistical significance was set at *p* < 0.05.

## Results

### Proactive Responses Decreased With Age

**Figure 1:**
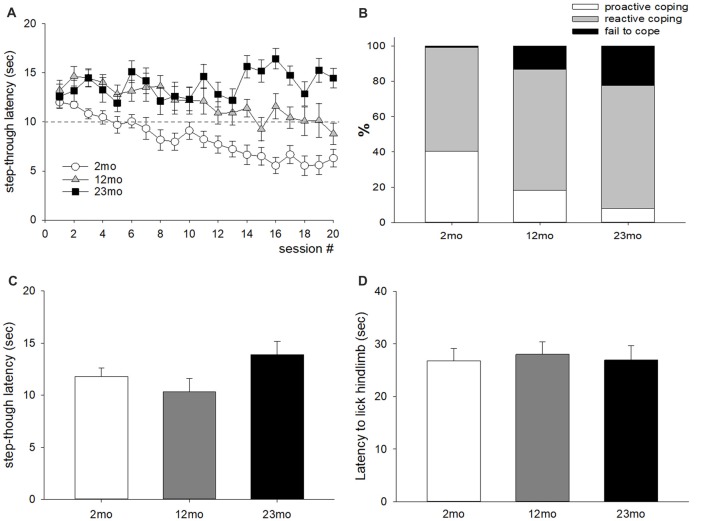
Age-related differences in coping style in the active avoidance test. **(A)** Step-through latency throughout 20 test sessions. Repeated measures ANOVA showed a significant training (within factor) effect (*F*_(19,874)_ = 4.74, *p* < 0.001), age (between factor) effect (*F*_(2,46)_ = 24.6, *p* < 0.001), and training by age interaction (*F*_(38,874)_ = 3.28, *p* < 0.001). *Post hoc* pairwise comparisons showed that step-through latency was longer for 12-month-old mice than 2-month-old mice (*p* < 0.001), and for 23-month-old mice than 12-month-old mice (*p* = 0.04). The dashed line indicates the time for electric foot shock presentation. **(B)** Percentiles of avoid (escape before shock presentation), escape (escape during shock presentation), and fail to escape during active avoidance training; these behaviors were considered proactive, reactive and fail to cope, respectively. A chi-square test of independence revealed a significant relationship between age and coping response ($\chi_{\left(4\right)}^{2}$ = 159.6, *n* = 980, *p* < 0.001). Proactive coping response decreased with increasing age. **(C)** Seventy-two hours delayed measurement of step-through latency. There was no significant difference between age groups. **(D)** Latency to lick the hind limb in the hot plate test. There was no significant difference between age groups. All data are the mean ± SEM. 2 mo: 2-month old (*n* = 20), 12 mo: 12-month old (*n* = 14), 23 mo: 23-month old (*n* = 15).

For the active avoidance test, mice that escaped before the presentation of foot shock were considered “proactive” and those that escaped after the presentation of foot shock were considered “reactive”. The average step-through latency was longer for 12-month-old mice than 2-month-old mice (*p* < 0.001), and for 23-month-old mice than 12-month-old mice (*p* = 0.04; Figure 1A). The ratio of proactive responses decreased with age (*p* < 0.001; Figure 1B). Step-through latency after 72 h did not differ by age (Figure 1C), indicating that age-related differences in memory did not affect the results of the experiment. In addition, no differences in licking time were observed between age groups in the hot plate test (Figure 1D), indicating that the difference in active avoidance did not depend on a difference in pain sensitivity.

**Figure 2:**
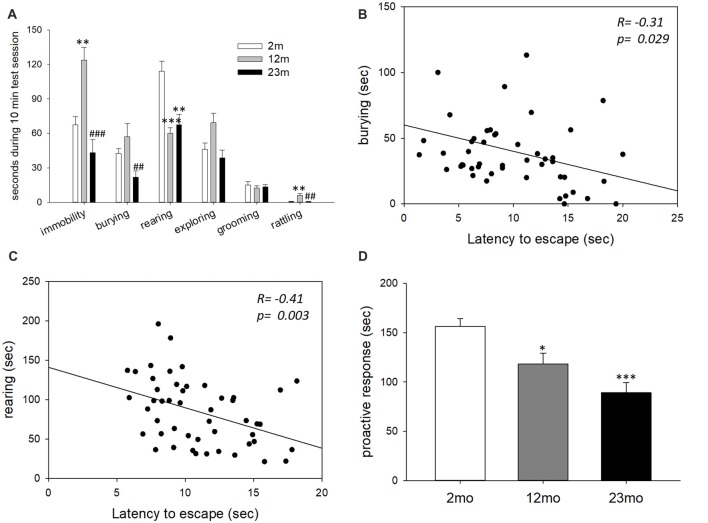
Age-related differences in coping style in the shock prod test. **(A)** Behavioral responses in the shock prod test. There were significant differences in immobility (*F*_(2,46)_ = 11.5, *p* < 0.001), burying (*F*_(2,46)_ = 5.7, *p* = 0.006), rearing (*F*_(2,46)_ = 12.3, *p* < 0.001) and rattling (*F*_(2,46)_ = 6.6, *p* = 0.003) by age group but not in exploring (*F*_(2,46)_ = 0.16, *p* = 0.86) and grooming (*F*_(2,46)_ = 0.08, *p* = 0.92). Immobility was higher in the 12-month-old mice than the 2-month-old mice (*p* = 0.003) and lower in the 23-month-old mice than the 12-month-old mice (*p* < 0.001). Burying response decreased in the 23-month-old mice compared to the 12-month-old mice (*p* = 0.005). Rearing response was reduced in the 12-month-old (*p* < 0.001) and 23-month-old (*p* = 0.001) mice compared to the 2-month-old mice. Rattling response increased in the 12-month-old mice compared to the 2-month-old mice (*p* = 0.006) but decreased in the 23-month-old mice compared to the 12-month-old mice (*p* = 0.009). **(B)** Correlation between the burying response in the shock prod test and escape latency in the active avoidance test. **(C)** Correlation between the rearing response in the shock prod test and escape latency in the active avoidance test. **(D)** Time of proactive responses (burying + rearing) in the shock prod test. There were significant differences between age groups (*F*_(2,46)_ = 13.5, *p* < 0.001). Pairwise comparisons showed a significant decrease in proactive responses in the 12-month-old (*p* = 0.017) and 23-month-old (*p* < 0.001) mice compared to the 2-month-old mice. All data are the mean ± SEM. 2 mo: 2-month old (*n* = 20), 12 mo: 12-month old (*n* = 14), 23 mo: 23-month old (*n* = 15). *p < 0.05, **p < 0.01, ***p < 0.001 vs. 2 mo, ^##^*p* < 0.01, ^###^*p* < 0.001 vs. 12 mo. ANOVA, Tukey’s HSD *post hoc* test **(A,D)**. Pearson correlation **(B,C)**.

In the shock prod test, immobility time was higher for 12-month-old mice than 2-month-old mice (*p* = 0.003) and lower for 23-month-old mice than 12-month-old mice (*p* < 0.001). Burying time was shorter in 23-month-old mice than 12-month-old mice (*p* = 0.005). Rearing time was shorter in 12-month-old (*p* < 0.001) and 23-month-old (*p* = 0.001) mice than 2-month-old mice. Rattling time was longer in 12-month-old mice than 2-month-old mice (*p* = 0.006) but was shorter in 23-month-old mice than 12-month-old mice (*p* = 0.009; Figure 2A). By correlation analysis, burying (*p* = 0.029) and rearing (*p* = 0.003) were considered proactive responses because they showed a significant negative correlation with step-through latency in the active avoidance test (Figures 2B,C). The total time for proactive responses (burying + rearing) in the shock prod test decreased in 12-month-old (*p* = 0.017) and 23-month-old (*p* < 0.001) mice compared to 2-month-old mice (Figure 2D).

### Stress Reactivity Decreased With Age

**Figure 3:**
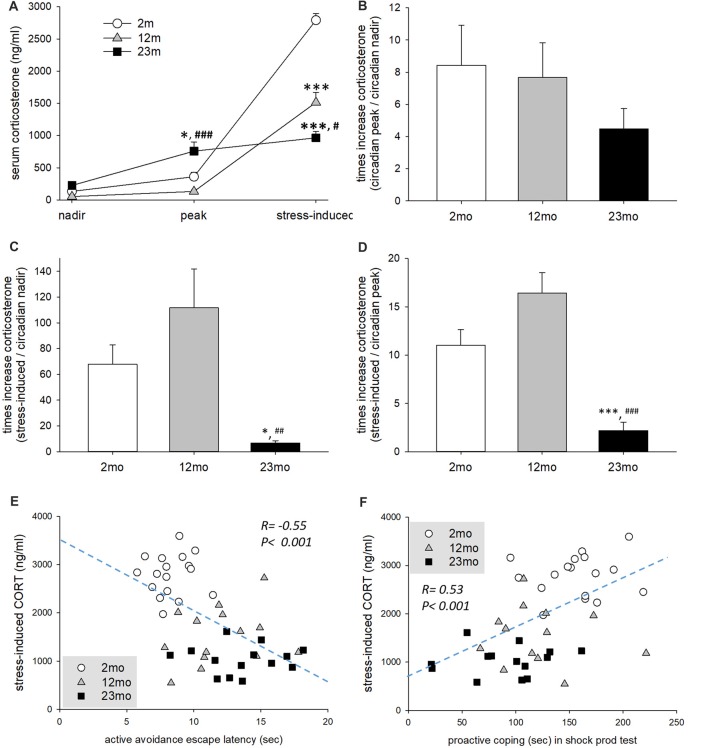
Age-related differences in stress reactivity in relation to coping style. **(A)** Serum corticosterone concentration at circadian nadir and peak and after immobilization stress. Repeated measures ANOVA revealed a significant within-subject effect (*F*_(2,84)_ = 356.6, *p* < 0.001), between-subject effect (*F*_(2,42)_ = 17.8, *p* < 0.001) and within-between interaction (*F*_(4,84)_ = 60.5, *p* < 0.001). By pairwise comparison, the circadian peak corticosterone concentration of 23-month-old mice was higher than 2-month-old (*p* = 0.017) and 12-month-old (*p* < 0.001) mice. However, immobilization stress-induced corticosterone concentration of 12-month-old (*p* < 0.001) and 23-month-old (*p* < 0.001) mice was lower than 2-month-old mice. In addition, 23-month-old mice had lower immobilization stress-induced corticosterone concentration than 12-month-old mice (*p* = 0.02). **(B)** Fold change of circadian peak vs. circadian nadir corticosterone concentration. There was no significant difference among age groups (*F*_(2,38)_ = 1.0, *p* = 0.376). **(C)** Fold change of stress-induced vs. circadian nadir corticosterone concentration. There was a significant group difference (*F*_(2,38)_ = 7.2, *p* = 0.002), and fold change was decreased in 23-month mice compared to 2-month (*p* = 0.045) and 12-month (*p* = 0.002) mice. **(D)** Fold change of stress-induced vs. circadian peak corticosterone concentration. There was a significant group difference (*F*_(2,38)_ = 19.2, *p* < 0.001), and fold change was decreased in 23 month mice compared to 2 month and 12 month (*p* < 0.001) mice. **(E)** Correlation of escape latency in active avoidance test and stress-induced corticosterone concentration. Pearson’s correlation coefficient was −0.55 (*p* < 0.001).** (F)** Correlation between proactive coping behaviors in the shock prod test and stress-induced corticosterone concentration. The Pearson correlation coefficient was 0.53 (*p* < 0.001). All data are the mean ± SEM. 2 mo: 2-month old (*n* = 17), 12 mo: 12-month old (*n* = 14), 23 mo: 23-month old (*n* = 15). *p < 0.05, ***p < 0.001 vs. 2 mo, ^#^*p* < 0.05, ^##^*p* < 0.01, ^###^*p* < 0.001 vs. 12 mo.

The concentration of serum corticosterone measured at the circadian nadir did not differ among age groups, but circadian peak concentration was higher for 23-month-old mice than 2-month-old (*p* = 0.017) and 12-month-old (*p* < 0.001) mice. Immobilization stress-induced corticosterone concentration was lower for 12-month-old (*p* < 0.001) and 23-month-old mice than 2-month-old mice (*p* < 0.001). In addition, 23-month-old mice had lower immobilization stress-induced corticosterone concentration than 12-month-old mice (*p* = 0.02; Figure 3A). Stress reactivity was estimated by the calculation of fold changes between circadian nadir, circadian peak and stress-induced corticosterone concentration. There was no difference in the circadian peak to nadir concentration ratio between age groups (Figure 3B). But the stress-induced to circadian nadir concentration ratio was decreased in 23-month (2 month vs. 23 month, *p* = 0.045; 12 month vs. 23 month, *p* = 0.002) mice (Figure 3C). Also the stress-induced to circadian peak concentration was decreased in 23-month (*p* < 0.001) mice (Figure 3D). Stress-induced corticosterone concentration was inversely proportional to both the step-through latency in the active avoidance test and the length of proactive responses in the shock prod test (Figures 3E,F). This indicates that the higher the concentration of stress-induced corticosterone, the more proactive was the individual.

### The Effect of Age, Stress Reactivity and Coping Style on Conditioned Fear Response

**Figure 4:**
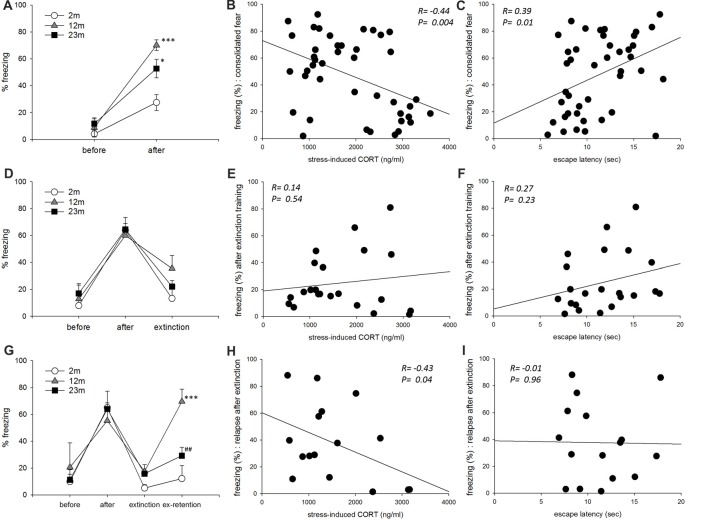
The influence of age, stress reactivity and coping style on conditioned fear. **(A)** Conditioned contextual fear consolidation. Repeated measures ANOVA showed a significant before-after training effect (*F*_(1,39)_ = 78.9, *p* < 0.001), age effect (*F*_(2,39)_ = 11.2, *p* < 0.001), and training by age interaction (*F*_(2,39)_ = 5.1, *p* = 0.011). By pairwise comparisons, 12-month-old (*p* < 0.001) and 23-month-old (*p* = 0.011) mice froze more often than 2-month-old mice (2 month, *n* = 17; 12 month, *n* = 11; 23 month, *n* = 14). **(B)** Correlation between stress-reactivity (stress-induced corticosterone concentration) and conditioned fear consolidation. **(C)** Correlation between coping style (escape latency in the active avoidance test) and conditioned fear consolidation. **(D)** Conditioned fear extinction. Only mice that froze more than 50% after training were included. Repeated measures ANOVA revealed a significant before-after training effect (*F*_(2,38)_ = 34.7, *p* < 0.001) but no significant age effect (*F*_(2,19)_ = 0.59, *p* = 0.57) or training by age interaction (*F*_(4,38)_ = 0.86, *p* = 0.47) (2 month, *n* = 5; 12 month, *n* = 8; 23 month, *n* = 9). **(E)** Correlation between stress-reactivity (stress-induced corticosterone concentration) and conditioned fear extinction. **(F)** Correlation between coping style (escape latency in the active avoidance test) and conditioned fear extinction. **(G)** Conditioned fear extinction retention. Only mice that froze less than 20% after extinction training were included. Repeated measures ANOVA revealed a significant before-after training effect (*F*_(3,39)_ = 23.9, *p* < 0.001), age effect (*F*_(2,13)_ = 4.37, *p* = 0.035) and training by age interaction (*F*_(6,39)_ = 3.13, *p* = 0.013). By pairwise comparisons, 12-month-old mice froze more than 2-month-old mice (*p* = 0.001), and 23-month-old mice froze less than 12-month-old mice (*p* = 0.007) (2 month, *n* = 4; 12 month, *n* = 5; 23 month, *n* = 7). **(H)** Correlation between stress-reactivity (stress-induced corticosterone concentration) and relapse of extinct fear. **(I)** Correlation between coping style (escape latency in the active avoidance test) and relapse of extinct fear. All data are the mean ± SEM. 2 mo: 2-month old, 12 mo: 12-month old, 23 mo: 23-month old. *p < 0.05, ***p < 0.001 vs. 2 mo, ^##^*p* < 0.01 vs. 12 mo.

In the fear conditioning paradigm, freezing as a measurement of consolidated fear memory was higher in 12-month-old (*p* < 0.001) and 23-month-old (*p* = 0.011) mice than 2-month-old mice (Figure 4A). Freezing was negatively correlated with stress-induced corticosterone concentration and positively correlated with escape latency in the active avoidance test (Figures 4B,C). As a result of frequency analysis, percent freezing by fear acquisition showed two peaks in the range of 10–20 and 60–70, and the lowest frequency in the range of 30–50. Therefore, mice freezed greater than 50% were selected for further analysis. In these mice, age was not associated with the extinct fear response (Figure 4D). Freezing after extinction training was not correlated with stress-induced corticosterone concentration or with escape latency in the active avoidance test (Figures 4E,F). As a result of frequency analysis, percent freezing after extinction training was mostly distributed below 20 but was rarely observed in other sections. Therefore, mice with a fear response lessor than 50% were selected for further analysis. In these mice with a consolidated fear response greater than 50% and post-extinction fear response less than 20%, relapse of fear was higher in 12-month-old mice than 2-month-old mice (*p* < 0.001), but lower in 23-month-old mice than 12-month-old mice (*p* = 0.007; Figure 4G). Freezing by fear relapse was negatively correlated with stress-induced corticosterone concentration but was not correlated with escape latency in the active avoidance test (Figures 4H,I).

### Age Differences in the Hippocampal Expression of Corticosterone Response Regulatory Proteins

**Figure 5:**
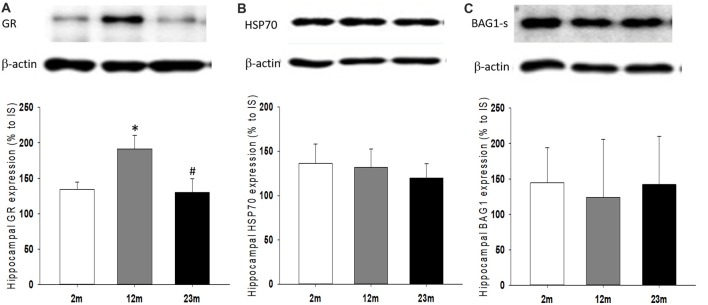
Age differences in hippocampal expression of glucocorticoid receptor (GR) and regulatory co-chaperones. **(A)** Relative expression of GR. ANOVA showed a significant age group difference (*F*_(2,33)_ = 4.1, *p* = 0.025). By pairwise comparisons, GR expression increased in 12-month-old mice (2 month vs. 12 month, *p* = 0.039) and decreased in 23-month-old mice (12 month vs. 23 month, *p* = 0.04). **(B)** Relative expression of HSP70. **(C)** Relative expression of BAG-1s. All data are the mean ± SEM. 2 mo: 2-month old (*n* = 17), 12 mo: 12-month old (*n* = 11), 23 mo: 23-month old (*n* = 11). % to IS, percent to internal standard. *p < 0.05 vs. 2 mo, ^#^*p* < 0.05 vs. 12 mo.

As a feedback regulator of the HPA axis and stress hormone secretion, the expression of GR and its regulatory co-chaperones were measured. The hippocampal expression of GR protein was higher in 12-month-old mice than 2-month-old mice (*p* = 0.039) and lower in 23-month-old mice than 12-month-old mice (*p* = 0.04; Figure 5A). There were no significant age-related differences in Hsp70 and Bag1-s expression (Figures 5B,C).

## Discussion

The difference in stress responsiveness and coping strategy by age is an important starting point for the understanding and prevention of stress-related diseases. We compared stress coping behaviors and corticosterone levels in three different age groups of male mice. The results revealed that aged mice showed decreased stress responsiveness and proactivity in coping responses. Consolidation and recurrence of conditioned fear memory appeared highest in the middle-aged mice, which may be related to hippocampal GR expression.

Coping behaviors were measured by two behavioral tests. In the active avoidance test, mice that moved into a second compartment before the presentation of electric shock showed a response of “avoid,” which was considered a proactive coping response. Moving into the second compartment after the presentation of electric shock was defined as “escape” and considered a reactive coping response. The step-through latency increased with age, indicating that proactivity in coping response decreased with age. Although differences in memory or pain thresholds could lead to differences in the step-through latency, there were no measurable differences between age groups in step-through latency 72 h later in the active avoidance test chamber. There were also no differences in pain sensitivity in the hot plate test.

The shock prod test revealed a similar pattern of coping style changes by age. The test measured whether responses to an electrified rod were either active or passive. Burying is considered an active coping reaction that is an attempt to bury dangerous objects, and immobility is a passive reaction to avoid danger (Diamant et al., 1991). The time spent burying and rearing was negatively correlated with the latency to escape in the active avoidance test and was considered a proactive response. The total time spent burying and rearing gradually decreased with age, especially the rearing time was lower in 12-month and 23 month than 2-month-old mice, and burying time was lower in 23-month-old mice than other aged groups. These two behavioral responses may be related to proactive coping but controlled by other mechanisms. In human studies, young people tend to actively respond and cope with stressors, but older people tend to ignore and avoid the stressors (Folkman et al., 1987). It has also been shown that middle-aged and older people have less hostile responses to stressors than younger ones (McCrae, 1982).

Circulating concentration of corticosterone, the main type of stress hormone in rodents, fluctuates with the circadian rhythm. Being nocturnal animals, mice have the lowest concentration of corticosterone in the morning (circadian nadir) and highest in the evening (circadian peak). A comparison of the corticosterone concentration of different age groups revealed no differences at the circadian nadir; however, at the circadian peak, 23-month-old mice showed the highest concentration. An increase in circulating stress hormone concentration by age also occurs in humans (Lupien et al., 1998). However, stress hormone concentration after immobilization stress revealed a different pattern. It was highest in 2-month-old mice and lowest in 23-month-old mice. These findings suggest that young mice usually maintain low concentrations of stress hormone, but on demand, they can produce a large amount of stress hormone. In contrast, older mice usually have high concentrations and do not produce much more when this hormone is needed. A previous study has shown that aged mice have delayed recovery of glucocorticoids to baseline after stress exposure, which may contribute to an increase in baseline corticosterone concentration (Nicolson et al., 1997). In humans, basal cortisol levels increase with age, which appears to be due to a weakening of homeostatic regulation, but not by the response to a stressor that increases in magnitude or duration during normal human aging (Nicolson et al., 1997). Similarly, meta-analysis of five studies showed that the ACTH response to stress is higher in younger than older adults (Kudielka et al., 2004).

Next, the influence of age, stress reactivity and coping style on fear regulation was measured with the fear conditioning paradigm. In humans, glucocorticoid hormones facilitate the long-term storage of fear memory (Zorawski and Killcross, 2002). In rats, a deficit of glucocorticoid hormone by adrenalectomy impairs the consolidation of contextual fear (Pugh et al., 1997). The maintenance of the normal function of the hormone receptor GR is also important because blunting GR signaling disrupts the long-term consolidation of fear memory (Rodrigues and Sapolsky, 2009). In our study, 12-month-old mice had the highest rate of fear memory consolidation and relapse of extinct memory. Although 2-month-old mice had the highest increase in stress-induced corticosterone concentration, 12-month-old mice had the highest increase in the ratio of stress-induced to circadian peak corticosterone concentration. In addition, the hippocampal expression of GR was highest in this age group.

Second, regarding the intensity of the stress response, previous research has found that animals that display less stress response to novelty are more susceptible to conditioned fear (Cordero et al., 2003). Our findings also indicated that when lower hormone concentration was associated with greater fear memory formation. However, 23-month-old mice showed a lower mean value of stress-induced corticosterone concentration than 12-month-old mice, suggesting that the formation of fear memory should be highest at 23 months. The 12-month-old mice may have shown the highest rate of fear memory formation because of age-related differences in GR expression in the hippocampus. Previous research suggests that activation of GR can enhance fear memory by the involvement in fear memory reconsolidation (Meir Drexler and Wolf, 2017), and GR blockade can inhibit the fear memory reinstatement (Pitman et al., 2011). If the amount of GR expression is more influential than the stress hormone concentration itself, our findings for fear memory formation at 12 months are explained. The higher prevalence of post-traumatic stress disorder in people in their 40–50 s may be explained by a similar mechanism (Ditlevsen and Elklit, 2010).

Extinction of fear memory was measured in a subgroup of mice that showed consolidated fear-induced freezing of greater than 50%. As a result, the degree of fear memory erasure did not differ among the age groups. In addition, extinction of fear memory did not correlate with stress responsiveness or coping style. The extinction of fear memory is known to be due to the excitement of intercalated cell masses of the amygdala through the discharge of the infralimbic prefrontal cortex (Myers and Davis, 2007). Age-related changes in these pathways are poorly understood. Another system that is important in fear memory extinction is the endocannabinoid system (Milad and Quirk, 2012). Deletion of the CB1 cannabinoid receptor increases passive coping tendency (Metna-Laurent et al., 2012). However, a study that used conditional knockouts found that an increase in passive coping was specific to the knockout of glutamatergic neurons, while GABAergic neuron selective knockout was associated with active coping. As there was no difference in the rate of extinction of fear memory between the age groups in our study, as well as no correlation between stress responsiveness and coping style, the infralimbic cortical pathway or endocannabinoid system does not seem to be affected by aging.

Extinction retention of fear memory was measured in a subgroup of mice that showed freezing of less than 20% after extinction training. Extinct fear memory was retained in 2-month-old mice only, while 12-month-old mice showed a high rate of relapse. Mice with lower circulating corticosterone also showed a higher recurrence of fear memory. Coping style was not associated with recurrence. This indicates that the formation of fear memory may be associated with both stress response and coping, whereas the maintenance of extinct fear memory is associated with stress responsiveness only. Consolidation of extinction learning requires novel protein synthesis in the medial prefrontal cortex (Santini et al., 2004). D-cycloserine, a partial NMDA receptor agonist, improves extinction retention in rats when administered immediately after extinction training (McCallum et al., 2010). In addition to the glutamatergic system, the serotonergic system may also play an important role in the maintenance of extinct fear memory. A deficit in fear extinction retention is a phenotype of the serotonin transporter (5-HTT) gene defect, which is associated with increased vulnerability to stress and potentiated fear by deficit in extinction retention, but not in fear consolidation and extinction (Wellman et al., 2007). The short variant of the 5-HTT promotor polymorphism that causes reduced activity of 5-HTT is associated with increased emotional reactivity to fear-invoking stimulus and likelihood of depression (Wilhelm et al., 2007). Therefore, glutamatergic and serotonergic circuits associated with stress responsiveness in the prefrontal cortex are presumed to be related to the maintenance of fear memory.

The biological response to glucocorticoids is mediated by binding to two types of stress hormone receptors, the GR and mineralocorticoid receptor (MR). GR expression in the hypothalamus, pituitary, and hippocampus exerts a negative regulatory response to the HPA axis. As a cytoplasmic neurohormonal receptor, GR signaling is mediated by various co-chaperones, such as Hsp70 and BAG1. A previous study found that GR mRNA expression is higher in adolescents and adults than infants or the aged (Perlman et al., 2007). Although GR activity decreases with age, age-related changes in the expression of GR-regulating co-chaperones are unknown. We found that hippocampal expression of Hsp70 and BAG1s remained constant throughout age in mice. In addition, these co-chaperones were independent of age-related changes in stress responsiveness and coping style.

In association with the large-scale brain networks, activation of the salience network is suggested to be associated with proactive coping (Hermans et al., 2014). In the young mice, as the amplitude of stress-induced corticosteroid concentration was greater, the activity of the salience network may have been higher, which explains proactive behavior more prominent in young mice (Korte et al., 1992). On the other hand, in the aged mice, the amplitude gradually decreased which may have resulted in less activation of the salience network and less proactivity.

Saliency to external stimuli is important for the formation of fear memory. Early phase of the acute stress response triggers a sensory hypervigilant state accompanied by an increased reliance on rapid but more rigid stimulus-response behaviors such as classical conditioning (Luethi et al., 2008). But for conditioned fear consolidation, saliency may not be the only factor. The burying response in the shock prod test, which is a proactive behavior, is more pronounce when NE levels are high and corticosteroid level is low. But fear memory is more enhanced when NE level are low and corticosteroid are high (Sgoifo et al., 1996). This implies that when fear memory is formed, not only an increase in saliency but also other factors related to corticosteroid signaling work together. Administration of hydrocortisone has also been shown to reduce the sensory gating (Richter et al., 2011). Membrane bound GR increases the activity of NE and is also involved in the weakening of executive control by the prefrontal cortex in the acute phase of the stress response (Hermans et al., 2014). Because the GR expression was higher in 12 months, this may have caused higher fear consolidation in this age group. And when the GR expression decreased, the consolidated fear also decreased in 23-month mice. High GR activity may have enhanced the executive control network in the late stage of stress (Hermans et al., 2014), but this didn’t contribute to our findings because the measurement of fear response in the experiment was done at the acute period of stress.

In summary, circadian peak concentration of corticosterone gradually increased with age, but stress responsiveness was highest in middle-aged mice and lowest in old-aged mice. With regard to coping strategies, proactive responses gradually decreased with age. Consolidation of fear memory was highest in middle-aged mice in association with stress responsiveness and coping behaviors, but the retention of extinct fear memory was associated with stress responsiveness only. Quantitative changes in GR with age may be associated with stress responsiveness or changes in coping style but not with changes in Hsp70 or BAG1.

## Author Contributions

H-JO, MS, S-YC, YKK and MY contributed to the experiments. YK, J-RB, JYB, YL, JS and SM contributed to data processing. SM contributed to organizing and planning the research.

## Conflict of Interest Statement

The authors declare that the research was conducted in the absence of any commercial or financial relationships that could be construed as a potential conflict of interest.
